# Norepinephrine promotes glioma cell migration through up-regulating the expression of Twist1

**DOI:** 10.1186/s12885-022-09330-9

**Published:** 2022-02-26

**Authors:** Xue Wang, Ying Wang, Fang Xie, Zi-Tian Song, Zi-Qian Zhang, Yun Zhao, Shi-Da Wang, Hui Hu, Yan-Shu Zhang, Ling-Jia Qian

**Affiliations:** 1 Institute of Military Cognition and Brain Sciences, Academy of Military Medical Sciences, #27 Taiping Road, Haidian, Beijing, 100850 China; 2 Laboratory Animal Center, North China University of Science and Technology, Tangshan, 063210 Hebei China; 3 Qingdao Eighth People’s Hospital, Qingdao, 266041 Shandong Province China

**Keywords:** Norepinephrine, Glioma, Cell migration, Twist1

## Abstract

**Background:**

Glioma cells are characterized by high migration ability, resulting in aggressive growth of the tumors and poor prognosis of patients. It has been reported that the stress-induced hormone norepinephrine (NE) contributes to tumor progression through mediating a number of important biological processes in various cancers. However, the role of NE in the regulation of glioma migration is still unclear. Epithelial-to-mesenchymal transition (EMT) is one of the most important steps for tumor migration and metastasis. Twist1, as a key regulator of EMT, has been found to be elevated during glioma migration. But it is still unknown whether Twist1 is involved in the effect of NE on the migration of glioma cells.

**Methods:**

Wound healing assay and transwell assay were conducted to evaluate the migration of glioma cells upon different treatments. The mesenchymal-like phenotype and the expression of Twist1 after NE treatment were assessed by cell diameters, real-time PCR, western blot and immunofluorescence staining. The gain-and loss-of-function experiments were carried out to investigate the biological function of Twist1 in the migration induced by NE. Finally, the clinical significance of Twist1 was explored among three public glioma datasets.

**Results:**

In this study, our finding revealed a facilitative effect of NE on glioma cell migration in a β-adrenergic receptor (ADRB)-dependent way. Mechanistically, NE induced mesenchymal-like phenotype and the expression of Twist1. Twist1 overexpression promoted glioma cells migration, while knockdown of Twist1 abolished the discrepancy in the migration ability between NE treated glioma cells and control cells. In addition, the clinical analysis demonstrated that Twist1 was up-regulated in malignant gliomas and recurrent gliomas, and predicted a poor prognosis of glioma patients.

**Conclusions:**

NE enhanced the migration ability of glioma cells through elevating the expression of Twist1. Our finding may provide potential therapeutic target for protecting patients with glioma from the detrimental effects of stress biology on the tumor progression.

**Supplementary Information:**

The online version contains supplementary material available at 10.1186/s12885-022-09330-9.

## Background

Malignant glioma is the most common primary intracranial tumor, accounting for approximately 80% of central nervous system (CNS) malignancies [1]. At present, the treatment of glioma is still mainly based on surgery to remove the tumor, and supplemented by radiation therapy, chemotherapy and other comprehensive treatment methods [2]. However, despite these treatments, the median survival of the patients with glioblastoma multiforme (GBM), which is the most malignant glioma [3], is approximately only 1 to 2 years [4]. Strong migration ability is one of the most important characteristics of glioma cells, resulting in aggressive growth and metastasis of the tumors, thus it is difficult to be completely removed by surgery [5, 6]. Therefore, it is urgent to deeply investigate the regulatory mechanism of glioma migration and explore new therapeutic targets.

Patients with cancer suffer from persistent strong mental and physical stress, which causes adverse stress reactions of the body and affects clinical treatment and prognosis seriously. A large number of epidemiological investigations and experimental studies have shown that chronic stress is closely related to the occurrence and development of a variety of tumors [7–10]. Chronic stress can induce aberrantly persistent activation of the Sympathetic Nervous System (SNS), leading to the simultaneous elevation of catecholamines [11]. It has been reported that the stress-induced hormones, especially norepinephrine (NE), contribute to tumor progression through mediating a number of important biological processes, such as inducing anti-apoptosis activity and chemotherapy resistance, disrupting antitumor immunity and promoting angiogenesis [12–15]. In addition, propranolol, a β-adrenergic receptor (ADRB) antagonist, has been demonstrated to inhibit tumor growth and metastasis, which provides a new strategy for the treatment of tumors [16, 17]. However, the reports about the involvement and the regulatory mechanism of NE in glioma are still rare.

In the present study, we explored the role of NE in the regulation of glioma cell migration, and elucidated that NE promoted glioma cell migration through binding to ADRBs. Mechanistically, NE induced mesenchymal-like phenotype and Twist1 expression. Twist1 overexpression facilitated the glioma cell migration, while knockdown of Twist1 abrogated the effect of NE on glioma cell migration. Moreover, our data revealed that Twist1 was up-regulated in malignant gliomas and recurrent gliomas, and predicted a poor prognosis of glioma patients.

## Methods

### Cell culture and intervention

The glioma cell lines U251 and LN229 were purchased from Chinese National Infrastructure of Cell Line Resource. The two cell lines were authenticated by STR analysis. The cells were cultured in DMEM medium (Sigma, St. Louis MO, USA) containing 10% FBS (Gibco, NY, USA), 100 U/ml penicillin and 100 U/ml streptomycin (HyClone, L.A., USA). All cells were grown in humidified incubator with 5% CO_2_ at 37 °C.

Twist1 expressing lentiviral vector was constructed by inserting the coding sequence (CDS) of human Twist1 gene (NM000474) in lentiviral GV358 vector (Genechem, Shanghai, China) which contained 3 × flag and green fluorescent protein (GFP). The shRNA oligonucleotides (oligos) targeting Twist1 were synthesized by Genechem Co.,Ltd. (Shanghai, China). The sequences of the synthesized oligos were as follows: 5′-CCGGTCCGCAGTCTTACGAGGAGCTCTCGAGAGCTCCTCGTAAGACTGCGGATTTTTG-3′ (forward) and 5′-AATTCAAAAATCCGCAGTCTTACGAGGAGCTCTCGAGAGCTCCTCGTAAGACTGCGGA-3′ (reverse). After annealing, double-strand oligos were inserted to lentiviral GV248 vector (Genechem, Shanghai, China). To produce lentivirus, HEK-293 T cells were co-transfected with the lentiviral vector described above and packaging vectors psPAX2 and VSVG using lipo2000 (Invitrogen, California, USA). The shRNA control virus and overexpression control virus were purchased from Genechem Co.,Ltd. (Shanghai, China).

Stable transfectants of glioma cells expressing Twist1 or a shRNA against Twist1 were established as described previously [18]. Briefly, U251 and LN229 cells were dissociated with 0.5% trypsin and seeded into six-well plates, following by infected with Twist1 overexpression virus, Twist1 knockdown virus and their control virus, separately. Then the stable infectants were screened by puromycin for 2 weeks.

NE (Sigma, St. Louis MO, USA), ADRB agonist (Isoprenaline) (Selleck Chemicals, Houston, USA) and antagonist (Propranolol) (Sigma, St. Louis MO, USA) were dissolved in Dimethyl Sulfoxide (DMSO) (Sangon Biotech, Shanghai, China) at the concentration of 20 mmol/L. Then, 0.5 μl/mL of the regents were added into the culture medium for intervention. Especially, propranolol was added 1 h before NE. The final concentration of all drugs was chosen based on successful activation/inhibition in previous publications: NE (10 μmol/L), isoprenaline (10 μmol/L), propranolol (10 μmol/L) [19, 20]. An equal volume of DMSO was used as control. In detail, when the cells were treated with only one regent (NE, isoprenaline or propranolol alone), 0.5 μl/mL of DMSO was used as control. When the cells were treated with two regents (NE combined with isoprenaline or propranolol), 1 μl/mL of DMSO was used as control.

### Wound healing assay

The procedure of wound healing assay was based on previous study [21]. The glioma cells were seeded in 6-well plates and cultured until they reached confluence. A wound was then created by manually scraping the cell monolayer with a 200 μL pipette tip. The cultures were rinsed several times with medium to remove dislodged cells, and 2 mL of DMEM medium with 1% FBS containing compounds were added into each well. Cells that had migrated into the wound area were photographed at 0 h and 24 h. After exporting images, the boundaries of the wound were indicated by drawing two straight lines, and the distance between the two lines was measured using Image J as the wound width at 0 h (X0) and 24 h (X24). The travelled distance percentage was determined as (X0-X24)/X0 × 100%.

### Transwell assay

To investigate cell migration ability, transwell assay was conducted using a 24-well insert with 8 μm pores (Corning, NY, USA) as previously reported [22]. In total, 5 × 10^4^ cells were seeded into the upper chamber of a polycarbonate transwell filters in 200 μL serum-free DMEM medium. The lower chamber was added with DMEM medium containing 20% FBS as chemoattractant. NE (10 μmol/L) or equal volume DMSO was added into the upper chamber. The cells were incubating for 6 h. The chambers were fixed with 10% neutral formalin for 15 min following by removing the non-migratory cells with cotton swabs gently. The cells were then dyed with crystal violet (Beyotime, Shanghai, China). Images were acquired using a microscope (Olympus, Tokyo, Japan). Then the number of cells of each field was counted manually. Besides, the staining areas were measured using Image-Pro plus 6.0. Three independent experiments were conducted and the data was presented as the means ± SD.

### Real time PCR

Total RNA from glioma cells was extracted using TRIzol reagent (Invitrogen California, USA). Synthesis of cDNA was done using the M-MLV Reverse Transcriptase (Promega, Wisconsin, USA) according to the manufacturer’s instructions. Real-time PCR was performed using a LightCycler 96 Real-time PCR System (Roche, Basel, Switzerland) with TB Green Premix Ex Taq kit (TaKaRa, Kyoto, Japan). The β-actin was selected as the endogenous control in the assay. Relative levels of indicated genes were calculated by 2^−ΔΔCt^ method as reported previously [23]. The primer sequences are provided as following:

**Table:** 

*N-cadherin*	Forward (5′-3′)	AGGATCAACCCCATACACCA
	Reverse (5′-3′)	TGGTTTGACCACGGTGACTA
*Fibronectin1*	Forward (5′-3′)	CAGTGGGAGACCTCGAGAAG
	Reverse (5′-3′)	TCCCTCGGAACATCAGAAAC
*Vimentin*	Forward (5′-3′)	GAGAACTTTGCCGTTGAAGC
	Reverse (5′-3′)	TCCAGCAGCTTCCTGTAGGT
*Twist1*	Forward (5′-3′)	GGAGTCCGCAGTCTTACGAG
	Reverse (5′-3′)	TCTGGAGGACCTGGTAGAGG
*β-actin*	Forward (5′-3′)	AATCGTGCGTGACATTAAGGAG
	Reverse (5′-3′)	ACTGTGTTGGCGTACAGGTCTT

### Immunofluorescence staining

Glioma cells were treated with NE or DMSO for 24 h and then washed with TBS for 2 times. Immunofluorescence staining was conducted based on previously reported [24]. The cells were fixed in 4% paraformaldehyde for 15 min, and permeabilized with 0.4% Triton X-100 for 5 min. After washing with TBS for 3 times, the cells were blocked with PBS containing 10% goat FBS (Solarbio, Beijing, China) and 1% BSA for 1 h and subsequently incubated with rabbit anti-human Twist1 antibody (1:50, Proteintech, Chicago, USA) at 4 °C overnight. After washed with TBS, the cells were incubated with Rhodamine-conjugated goat anti-rabbit IgG antibody (1:100, Proteintech, Chicago, USA) at room temperature for 30 min and then counterstained with 4′,6-diamidino-2-phenylindole (DAPI) (Solarbio, BeiJing, China). Images were captured using a fluorescence microscope (Olympus, Tokyo, Japan).

### Western blot analysis

Western blot analysis was performed as described previously [25]. The cells were lysed with Radio Immunoprecipitation Assay (RIPA) cell lysate (Solarbio, Beijing, China) containing protease inhibitors, and centrifuged at 12,000 g for 15 min at 4 °C to extract the supernatant. Then loading buffer (Tiangen, Beijing, China) was added and boiled for 5 min to denaturation. The samples were electrophoretic in 10% SDS-PAGE gel, following by transferred to poly vinylidene fluoride (PVDF) membrane using wet transfer system (Bio-rad, California, USA). The membrane was sealed in 5% milk at room temperature for 1 h and washed 3 times in 1 × TBST. The membrane was cut prior to hybridisation with antibodies. The primary antibodies were added separately and incubated overnight at 4 °C. After washing 3 times in 1 × TBST, the HRP labeled secondary antibodies were incubated for 2 h at room temperature. The membranes were prepared with ECL Western blotting substrate Kit (Thermo Scientific, Massachusetts, USA) followed with image acquisition by Image Quantlas 4000 (GE, NY, USA) and analysis by Image Quantlas 4000 and Image J. The original images of the blots shown in the figures could be found in the Supplementary Information file. The antibodies used in the study were Rabbit anti-Fibronection (1:500, Proteintech, Chicago, USA), N-cadherin (1:1000, Proteintech, Chicago, USA), Vimentin (1:3000, Proteintech, Chicago, USA), mouse anti β-actin (1:5000, Proteintech, Chicago, USA), Goat anti rabbit IgG (1:5000, ZSGB-Bio, Beijing, China) and Goat anti mouse IgG (1:5000, ZSGB-Bio, Beijing, China).

### Patient datasets and clinical analysis

The Glioma French dataset [26] and the cancer genome atlas (TCGA) dataset used in this study were publicly available from the R2: Genomics Analysis and Visualization Platform (http://r2.amc.nl). The expression of Twist1 in the two datasets and the survival analysis of patients from the TCGA dataset were also analyzed using this platform. In addition, the Chinese Glioma Genome Atlas (CGGA) dataset (mRNAseq_693) [27, 28] used in this study was collected and analyzed at CGGA (http://www.cgga.org.cn/).

### Data analysis

Statistical analysis was processed with SPSS 23.0 software. The data was expressed as mean ± SD. The continuous variables were evaluated for normality before comparison for statistical differences. Student’s t-test was used for comparison between two groups. One-way ANOVA analysis was performed to compare the values among multiple groups. Signifcant results of ANOVA were subjected to Bonferroni post hoc test. All differences were two-sided. A *p*-value less than 0.05 was considered to have significant difference.

## Results

### NE promoted glioma cells migration in an ADRB-dependent way

**Fig. 1:**
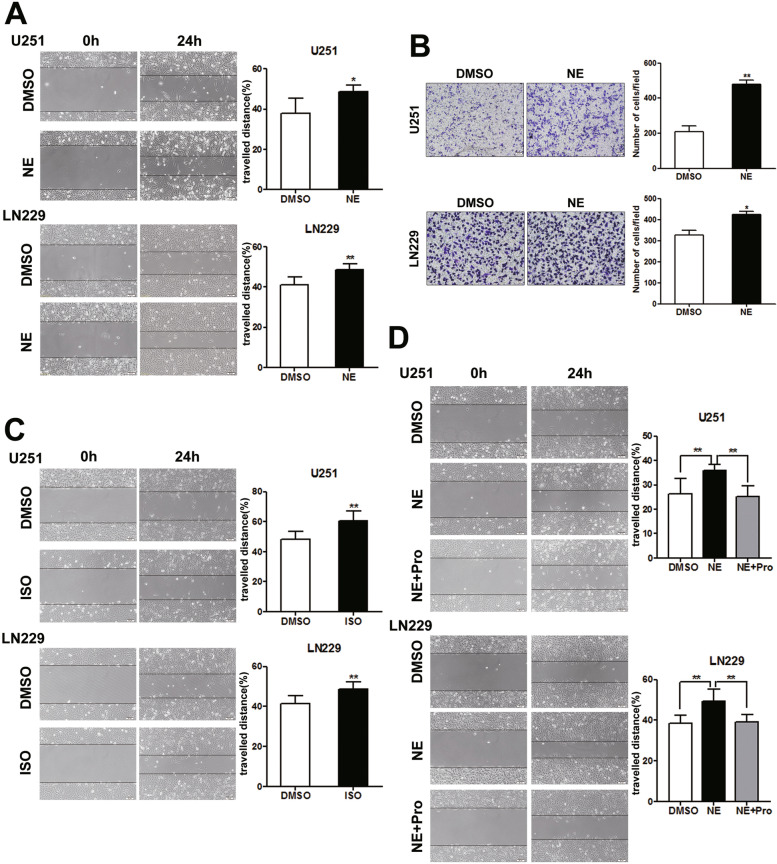
NE promoted glioma cells migration via ADRB. Wound healing assays and transwell assays were used to evaluate the effects of NE administration on glioma cells migration. **A** Representative images and the higher travelled distances of wound healing in NE-treated U251 (*n* = 4, *p* = 0.043) and LN229 (*n* = 4, *p* = 0.008) cells. Scale bar, 50 μm. Student’s t-test was used for statistical analysis. **B** Representative images and the increased cell number of transwell assays in NE-treated U251 (*n* = 3, *p* = 0.002) and LN229 (*n* = 3, *p* = 0.041) cells. Scale bar, 50 μm. Student’s t-test was used for statistical analysis. **C** Representative images and isoprenaline (ISO, 10 μmol/L) elevated the migration ability of U251 (*n* = 6, *p* = 0.002) and LN229 (*n* = 6, *p* = 0.006). Scale bar, 50 μm. Student’s t-test was used for statistical analysis. **D** Representative images and propranolol (Pro, 10 μmol/L) administration reversed the effect of NE on migration of U251 (*n* = 6, *p* = 0.002) and LN229 (*n* = 6, *p* = 0.001). Scale bar, 50 μm. One-way ANOVA analysis was used for statistical analysis. * *p* < 0.05, ** *p* < 0.01

The U251 and LN229 cells were treated with NE to investigate the effect of stress hormone on glioma cell behavior in vitro. Wound healing assay was used to examine the cell migration ability. Compared with the control group, the rates of travelled distance in the cells after NE treatment for 24 h were elevated (Fig. 1A, Student’s t-test, U251, *p* = 0.043; LN229, *p* = 0.008). Consistently, the results of transwell assay also showed that both the number of migrated cells (U251, *p* = 0.002; LN229, *p* = 0.041) and the staining area (U251, p = 0.002; LN229, *p* = 0.033) were increased upon NE treatment (Fig. 1B & Supplementary Fig. 1A, Student’s t-test). Studies have found that NE primarily binds to ADRB to exert its biological effect in various cancers such as ovarian, prostate and pancreatic cancer [10, 12, 29]. Isoprenaline, an ADRB agonist, was further utilized to evaluate the role of ADRB in glioma cells migration. The data of wound healing assay revealed that isoprenaline elevated the migration ability of U251 (*p* = 0.002) and LN229 (*p* = 0.006) (Fig. 1C, Student’s t-test). Besides, isoprenaline combined with NE could also promote the migration of glioma cells (Supplementary Fig. 1B, Student’s t-test, U251, *p* = 0.004; LN229, *p* = 0.007). Furthermore, the ratio of isoprenaline+NE/DMSO was increased compared with that of NE/DMSO or isoprenaline/DMSO in the travelled distance, suggesting a stronger role of isoprenaline combined with NE in the comparison with NE or isoprenaline alone in the migration of glioma cells (Supplymentary Fig. 1C, One-way ANOVA, U251, *p* = 0.0001; LN229, *p* = 0.003). In addition, an ADRB antagonist propranolol was demonstrated to restrain the migration triggered by NE (Fig. 1D, One-way ANOVA, U251, *p* = 0.002; LN229, *p* = 0.001), although the propranolol alone showed no obvious effect on the cells migration (Supplementary Fig. 1D, Student’s t-test, U251, *p* = 0.449; LN229, *p* = 0.892). Thus, our findings indicated that the stress hormone NE promoted glioma cells migration in an ADRB-dependent way.

### NE induced mesenchymal-like phenotype of glioma cells

**Fig. 2:**
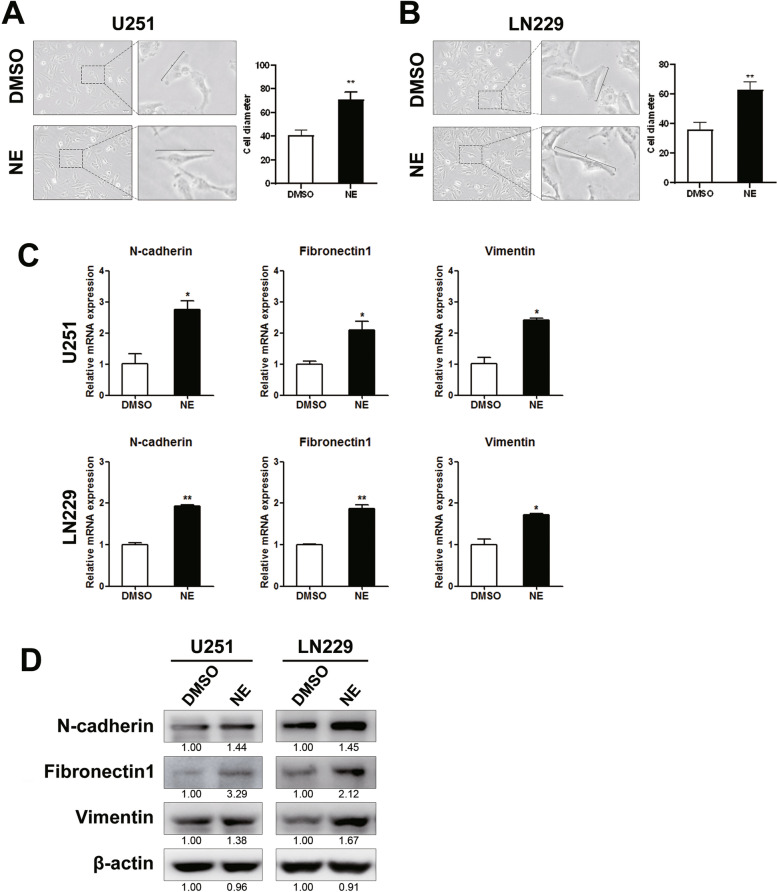
NE induced mesenchymal-like phenotype of glioma cells. The cell morphology and the levels of mesenchymal markers were assessed upon NE treatment. **A**-**B** The morphologies and the increased cellular diameters in NE-treated U251 (A, *n* = 20, *p* = 0.0001) and LN229 cells (**B**, *n* = 20, *p* = 0.0001) were shown. Scale bar, 20 μm. Student’s t-test was used for statistical analysis. **C** Real-time PCR analysis revealed elevated mRNA levels of N-cadherin (U251, *p* = 0.027; LN229, *p* = 0.003), Fibronectin1 (U251, *p* = 0.031; LN229, *p* = 0.006) and Vimentin (U251, *p* = 0.012; LN229, *p* = 0.018) in NE-treated U251 (*n* = 3) and LN229 (*n* = 3) cells. Student’s t-test was used for statistical analysis. **D** Western blot assay showed a various increase on the protein levels of these mesenchymal markers with NE administration. The relative levels of protein were marked as the mean number underlying the band images. * *p* < 0.05, ** *p* < 0.01

The epithelial-to-mesenchymal transition (EMT) is a critical event in mediating tumor migration, invasion and metastasis. Whether NE could regulate the EMT phenotype thus promote the migration of glioma cells is still unknown. EMT is characterized by cell morphologic changes from irregular polygons to elongated shape. So we determined the cell diameters after NE treatment. As shown in Fig. 2A & B, the cell diameters were significantly increased upon NE treatment compared with the control group (Student’s t-test, U251, *p* = 0.0001; LN229, p = 0.0001). Moreover, the results of real-time PCR assay showed that compared with DMSO group, the mRNA levels of mesenchymal markers including N-cadherin (U251, *p* = 0.027; LN229, *p* = 0.003), Fibronectin1 (U251, *p* = 0.031; LN229, *p* = 0.006) and Vimentin (U251, *p* = 0.012; LN229, *p* = 0.018) were up-regulated after NE intervention (Fig. 2C, Student’s t-test). Western blot results also revealed a various increase on the protein levels of N-cadherin, Fibronectin1 and Vimentin, which further confirmed a positive role of NE on the expression of mesenchymal markers (Fig. 2D & Supplementary Fig. 2). The aforementioned results suggested an induction of the mesenchymal-like phenotype by NE in U251 and LN229 cells.

### NE up regulated the expression of Twist1

**Fig. 3:**
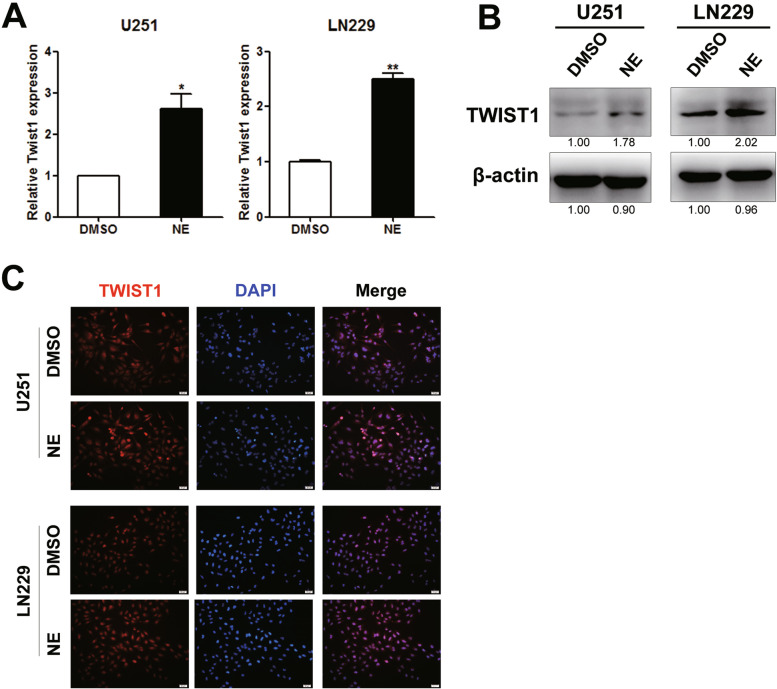
NE upregulated the expression of Twist1 in glioma cells. The effect of NE on the expression of Twist1 was investigated using real-time PCR, western blot and immunofluorescence staining. **A** Real-time PCR analysis revealed an up-regulation of Twist1 mRNA induced by NE administration in U251 (*n* = 3, *p* = 0.024) and LN229 (*n* = 3, *p* = 0.002). Student’s t-test was used for statistical analysis. **B** The facilitation of NE on Twist1 protein expression in U251 and LN229 cells. The relative levels of protein were marked as the mean number underlying the band images. **C** Immunofluorescence staining indicated an enhanced expression of Twist1 under NE treatment. The representative images were shown. * *p* < 0.05, ** *p* < 0.01

We further examined the influence of NE on Twist1 expression, which is one of the key regulators of EMT progress. As shown in Fig. 3A, the mRNA level of Twist1 was raised in NE-treated cells compared to the control cells (Student’s t-test, U251, *p* = 0.024; LN229, *p* = 0.002). Consistently, NE treatment also increased the expression of Twist1 protein (Fig. 3B & Supplementary Fig. 3A&B, Student’s t-test, U251, *p* = 0.007; LN229, *p* = 0.005). What’s more, the enhanced expression of Twist1 in the NE group was further validated by immunofluorescence staining (Fig. 3C).

### Twist1 facilitated mesenchymal-like phenotype and migration of glioma cells

**Fig. 4:**
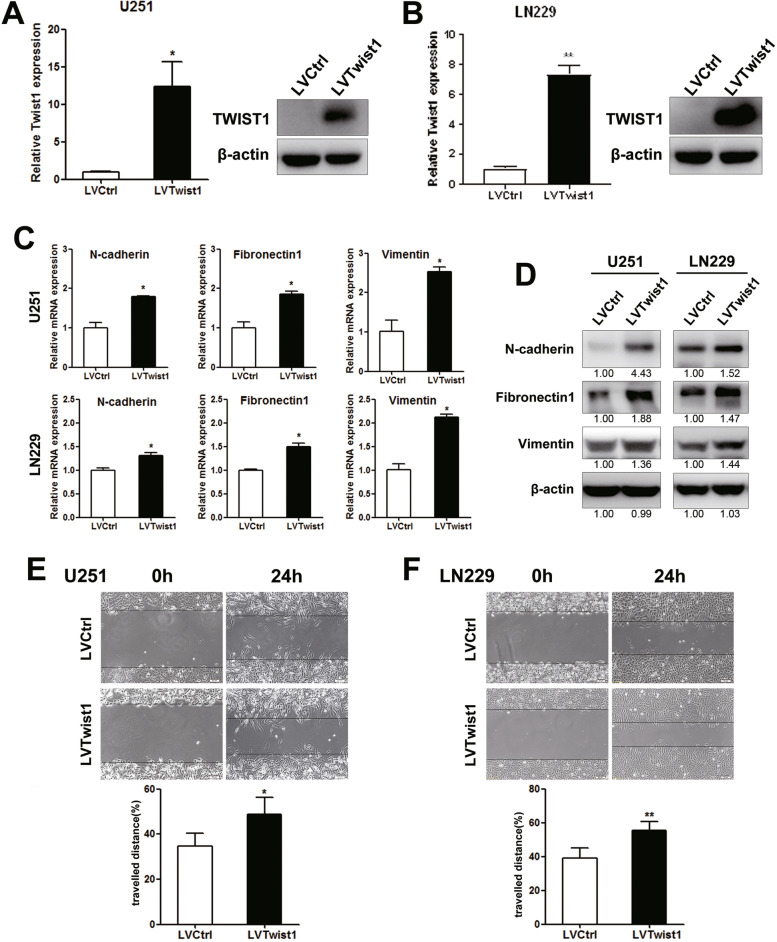
Twist1 overexpression facilitated mesenchymal-like phenotype and migration of glioma cells. Lentiviruses were used to establish the stable glioma cell lines with Twist1 overexpression. The levels of Twist1, mesenchymal markers and the migration ability were measured in the indicated cells. **A** The overexpression of Twist1 in established U251 cell lines were validated by real-time PCR (left panel, *n* = 3, *p* = 0.038) and western blot (right panel). Student’s t-test was used for statistical analysis. **B** The overexpression of Twist1 in established LN229 cell lines were validated by real-time PCR (left panel, *n* = 3, *p* = 0.005) and western blot (right panel). Student’s t-test was used for statistical analysis. **C** Real-time PCR revealed the increased mRNA levels of the mesenchymal markers in U251/LN229 LVTwist1 cells (U251: *n* = 3, N-cadherin *p* = 0.016, Fibronectin1 *p* = 0.014, Vimentin *p* = 0.021; LN229: *n* = 3, N-cadherin *p* = 0.011, Fibronectin1 *p* = 0.037, Vimentin *p* = 0.008). Student’s t-test was used for statistical analysis. **D** The higher expressed mesenchymal markers in LVTwist1 cells were shown. The relative levels of those markers were noted as the mean number underlying the band images. **E** Twist1 overexpression led to higher travelled distances of wound healing both in U251 (*n* = 5, *p* = 0.018) and LN229 cells (*n* = 5, *p* = 0.002). Scale bar = 50 μm. Student’s t-test was used for statistical analysis. *p < 0.05, **p < 0.01

Next, the U251 and LN229 cells were infected with Twist1 overexpression virus and control virus. The overexpression effect was confirmed by real-time PCR and western blot assay (Fig. 4A&B, Student’s t-test, U251, *p* = 0.038; LN229, *p* = 0.005). The role of Twist1 in regulating the migration of glioma cells was further explored. Compared with the control cells, Twist1 overexpression enhanced the mRNA levels of mesenchymal markers including N-cadherin (U251, *p* = 0.016; LN229, *p* = 0.011), Fibronectin1 (U251, *p* = 0.014; LN229, *p* = 0.037) and Vimentin (U251, *p* = 0.021; LN229, *p* = 0.008) (Fig. 4C, Student’s t-test). The results of western blot assay showed a various elevation on the protein levels of mesenchymal markers under Twist1 overexpression. Specifically, compared with the control cells, N-cadherin and Fibronectin1 increased significantly while Vimentin increased slightly in U251-LVTwist1 cells. And the protein levels of mesenchymal markers were promoted mildly in LN229-LVTwist1 cells (Fig. 4D). Moreover, the results of wound healing assay also revealed that Twist1 overexpression facilitated the migration of glioma cells (Fig. 4E&F, Student’s t-test, U251, *p* = 0.018; LN229, *p* = 0.002).

### Twist1 depletion restrained NE-induced glioma cells migration

**Fig. 5:**
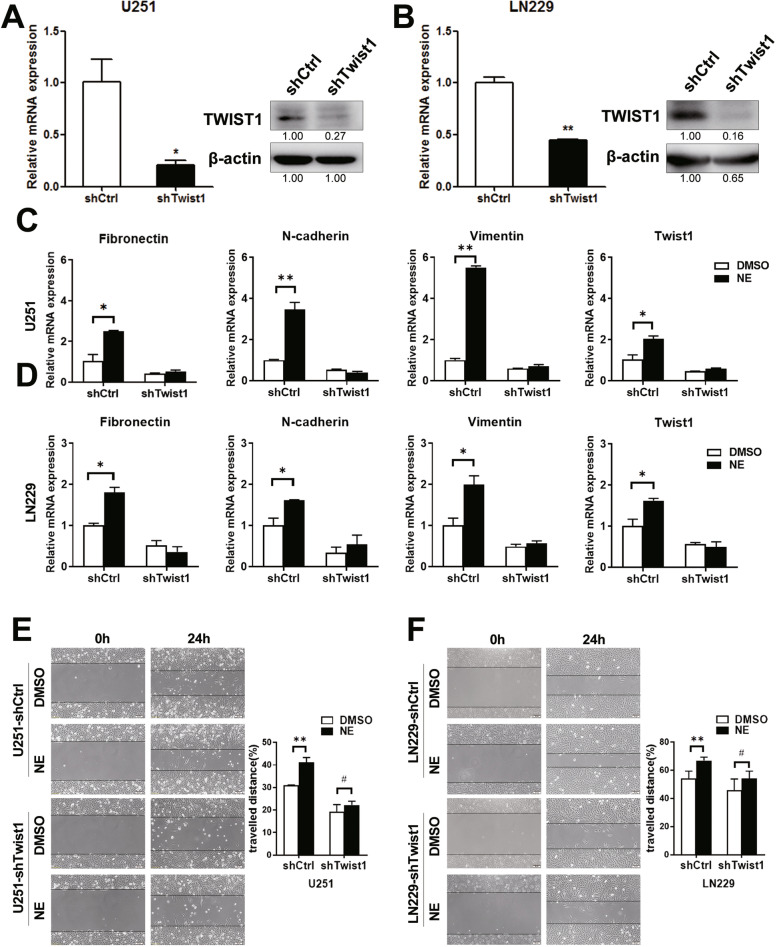
Twist1 knockdown suppressed NE-induced mesenchymal-like phenotype and migration of glioma cells. Lentiviruses were used to establish the stable glioma cell lines with shTwist1. The levels of Twist1, mesenchymal markers and the migration ability were detected in the indicated cells. **A**-**B** Real-time PCR (left panel) and western blot (right panel) were used to detect Twist1 expression. The mRNA and protein level of Twist1 significantly decreased in U251 (**A**, *n* = 3, *p* = 0.036) and LN229 cell lines (**B**, *n* = 3, *p* = 0.005). Student’s t-test was used for statistical analysis. **C**-**D** NE-induced upregulation of mesenchymal markers was blocked by shTwist1 lentivirus transfection both in U251 (**C**, *n* = 3, N-cadherin *p* = 0.014, Fibronectin *p* = 0.016, Vimentin *p* = 0.021) and LN229 cells (**D**, *n* = 3, N-cadherin *p* = 0.037, Fibronectin *p* = 0.011, Vimentin *p* = 0.008). One-way ANOVA analysis was used for statistical analysis. **E**-**F** Twist1 knockdown offset the pro-migration effects of NE in U251 (*n* = 5, *p* = 0.0001) and LN229 cells (*n* = 5, *p* = 0.001). One-way ANOVA analysis was used for statistical analysis. Scale bar = 50 μm, *p < 0.05, **p < 0.01

To further characterize the role of Twist1 in NE-mediated glioma cells migration, stable transfectants of glioma cells expressing shRNA against Twist1 were established. The knockdown effects were determined by real-time PCR and western blot assay (Fig. 5A&B & Supplementary Fig. 5, Student’s t-test, U251, *p* = 0.036; LN229, *p* = 0.005). As expected, knockdown of Twist1 alleviated the distinct expression levels of mesenchymal makers between glioma cells upon NE treatment and DMSO treatment (Fig. 5C&D, One-way ANOVA, U251, N-cadherin *p* = 0.014, Fibronectin *p* = 0.016, Vimentin *p* = 0.021; LN229, N-cadherin *p* = 0.037, Fibronectin *p* = 0.011, Vimentin *p* = 0.008). Consistently, Twist1 depletion also abrogated the discrepancy of migration between the NE treated glioma cells and the control cells (Fig. 5E&F, One-way ANOVA, U251, *p* = 0.0001; LN229, *p* = 0.001). Taken together, these results supported that knockdown of Twist1 inhibited glioma cells migration under NE treatment, indicating a regulative role of Twist1 in NE-mediated glioma cells migration.

### Twist1 was up-regulated in human gliomas and predicted a poor prognosis of glioma patients

**Fig. 6:**
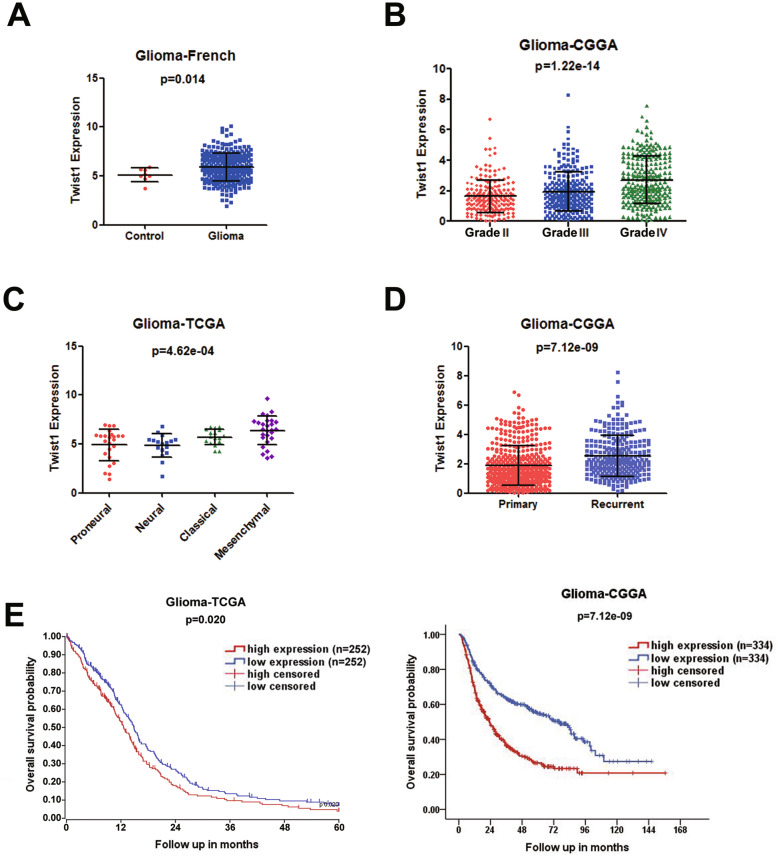
Expression and clinical correlation of Twist1 in glioma patients. The expression of Twist1 in clinical glioma samples and its relationship with patients’ survival time were analyzed in different glioma datasets. **A** A high level of Twist1 was observed in gliomas compared with control tissues from the French glioma database (*n* = 284, *p* = 0.014). Student’s t-test was used for statistical analysis. **B** The expression of Twist1 was increased from the WHO tumor grade II to grade IV in the CGGA glioma database (*n* = 692, *p* = 1.22e-14). One-way ANOVA analysis was used for statistical analysis. **C** Twist1 expression was up-regulated in mesenchymal subtypes compared to other indicated subtypes of glioma tumors from the TCGA glioma database (*n* = 170, *p* = 4.62e-04). One-way ANOVA analysis was used for statistical analysis. **D** Twist1 was hyper-expressed in the recurrent gliomas compared to the primary gliomas from the CGGA glioma database (*n* = 693, *p* = 7.12e-09). Student’s t-test was used for statistical analysis. **E** Kaplan-Meier survival analysis of patients from TCGA glioma database (*n* = 504, *p* = 0.02, left panel) and CGGA glioma database (*n* = 668, *p* = 7.12e-09, right panel) stratified by Twist1 expression. A lower survival probability in high Twist1 expressed patients compared with those patients with low Twist1 expression was revealed. Median Twist1 expression was used for stratification into Twist1 high expression and Twist1 low expression tumors

To explore the clinical significance of Twist1, we analyzed the levels of Twist1 in glioma tissues and normal tissues, different World Health Organization (WHO) tumor grades and different subtypes using multiple datasets. The result showed that the expression of Twist1 was higher in glioma samples than in control samples (Fig. 6A, Student’s t-test, *p* = 0.014). Besides, the expression of Twist1 was significantly increased from the WHO tumor grade II to grade IV, suggesting a correlation between the level of Twist1 and the malignant degree of glioma (Fig. 6B, One-way ANOVA, *p* = 1.22e-14). The analysis of the TCGA data set indicated that Twist1 was hyper-expressed in mesenchymal subtypes compared to proneural, neural and classical subtypes of glioma tumors (Fig. 6C, One-way ANOVA, *p* = 4.62e-04). As tumor migration has been found to be closely related to tumor relapse, we further analyzed the level of Twist1 in primary and recurrent gliomas. The result showed that Twist1 expression was up-regulated in the recurrent gliomas compared to the primary gliomas (Fig. 6D, Student’s t-test, *p* = 7.12e-09). In addition, the overall survival (OS) analysis demonstrated that Twist1 high expression in gliomas predicted short survival of patients by analyzing the TCGA database (Fig. 6E left & Supplementary Fig. 6, Kaplan-Meier method, *p* = 0.02 & 0.026, respectively) and CGGA datebase (Fig. 6E right, Kaplan-Meier method, *p* = 7.12e-09).

## Discussion

Malignant gliomas are highly infiltrative tumors, with cells migrating from the primary lesion into surrounding normal brain tissue, leading to complicating complete surgical resection, tumor recurrence and poor prognosis of patients. Thus, clarifying the underlying regulatory mechanism of glioma migration to identify novel targets for effective intervention is urgently needed [4]. In this study, our results showed that NE promoted glioma cells migration and induced mesenchymal-like phenotype. Furthermore, the biological effect of NE on glioma cells migration was attributed to the binding with ADRB and the regulation of Twist1 expression.

Epidemiological and experimental studies have shown that stress can modulate the progression of tumor, particularly, one large meta-analysis on 163,363 individuals published in 2017 revealed that self-reported psychological-distress scores are epidemiologically linked to cancer-related mortality [30]. However, the biological mechanisms underlying such effects are not well understood. Data from animal models has shown that stress could affect immune responses via neuroendocrine system [14]. Nevertheless, recent studies indicated that stress mediators from the sympathetic nervous system could also have a direct effect on the malignant behavior of tumor cells independent of the dysregulation on the immune system [31]. The catecholamine hormone, NE, is a crucial neurotransmitter released by the SNS in response to physiological, psychological, or environmental threats. It has been reported that NE could directly induce gene expression and was involved in invasion, migration, and metastasis in a variety of cancers [32–35]. However, little is known about the effect of NE on glioma cell migration. In this study, the data of wound healing assay showed that NE elevated the rate of travelled distance compared with the DMSO group. Consistently, the number of migrated cells was increased upon NE treatment evaluated by transwell assay. Our results suggested a promoting role of NE on glioma cells migration.

Families of adrenergic receptors mediate most of the biological effects of norepinephrine and can be divided into α- and β-adrenergic receptors. The growing evidence has suggested that ADRBs are predominantly responsible for the impact of NE and chronic stress on the progression of cancer [34]. Further, some studies have demonstrated that the expressions of ADRBs were higher in multiple types of tumor tissues compared with the corresponding normal tissues [36, 37]. Chronic stress and NE can accelerate tumor cell proliferation, angiogenesis and metastasis via the ADRB-mediated activation of cAMP/PKA signaling and thus upregulating the level of VEGF, MMPs, the phosphorylation of Bad and many other signaling pathways [10, 35, 38, 39]. So far, a growing number of studies have supported the use of β-blockers in reducing the rates of progression of several solid tumors and prolonging survival of cancer patients [40–43]. Propranolol, as an ADRB antagonist, has been demonstrated to inhibit tumor growth and metastasis, making it a new alternative for cancer adjuvant chemotherapy. In the present study, we found that ADRB agonist isoprenaline could increase the migration capacity of U251 and LN229 cells. In further, propranolol could significantly reduce the migration of glioma cells induced by NE. Our findings suggested that β-adrenergic blockade might play a role in glioma treatment, especially for the cancer patients suffering from the detrimental effects of chronic stress.

Tumor dissemination and metastatic behavior account for the vast majority of cancer associated mortality. Epithelial tumors achieve this progressive state via EMT, which has been shown to be an important step for tumor migration and metastasis [44]. During EMT, tumor cells gradually lose intercellular contacts and undergo morphological changes from epithelial cells to mesenchymal-appearing cells. The decreased epithelial marker E-cadherin and the up-regulated mesenchymal markers such as N-cadherin, Vimentin, Fibronectin1 are believed to be hallmarks of EMT [45, 46]. Studies have shown that glioma cells can undergo EMT or EMT(−like) changes to gain the ability of metastasis [47, 48]. However, the majority of GBMs do not show intrinsic E-cadherin expression and the mesenchymal transition occurs independently from E-cadherin to N-cadherin shift [49]. Similarly, we also found that the levels of E-cadherin were extremely low in the two glioma cell lines used in this research as the Ct values were more than 30 in real-time PCR analysis. Nevertheless, our data showed that compared with the control group, the cell diameters were increased after NE treatment, indicating a change in cell morphology. Furthermore, the expression of mesenchymal markers including N-cadherin, Fibronectin1, Vimentin increased upon NE intervention, suggesting that NE could enforce the mesenchymal-like phenotype of glioma cells.

We also explored the molecular mechanism of NE regulated glioma cells migration. Members of the TWIST-family are demonstrated to be crucial EMT-activators. While a great number of studies has implicated that Twist1 is overexpressed in various carcinomas and plays a vital role in tumor initiation, stemness, angiogenesis, dissemination and chemoresistance [50]. More recently, emerging evidence has shown that elevated expression of Twist1 was observed during the glioma cell invasion and migration [51]. Therefore, in this study we examined the influence of NE on Twist1 expression and found that NE increased the expression of Twist1 in U251 and LN229. We further used lentivirus to modulate the expression of Twist1. Consistent with previous reports, we also proved that overexpression of Twist1 could promote glioma cells migration. More importantly, our data revealed that knockdown of Twist1 abolished the migration trigged by NE. However, the molecular mechanism underlying high level of Twist1 under NE intervention remains unclear, which is one of the limitations of this study. Studies have shown that the expression of Twist1 is modulated by an array of different upstream regulators via multiple pathways including JAK2/STAT3, GSK-3β/β-catenin, TGF-β/Smad, PKB/AKT and NF-κB [49, 52–54]. In another study of our team, we have found that PI3K/Akt signaling was activated upon NE administration in glioma cells [19]. As reported in other literatures, the expression of Twist1 is closely regulated by the PI3K/AKT signaling pathway in glioma and many other tumors [55–57]. These findings suggest that the PI3K/Akt pathway may play a regulate role in the NE-induced Twist1 promotion, which needs more investigation.

## Conclusions

In summary, we have found that stress-induced hormones NE promoted glioma cell migration. Mechanistically, NE mainly bound to ADRB and further increased the expression of Twist1 to induced mesenchymal-like phenotype of glioma cells. Our findings may provide potential therapeutic targets and pave the way for the development of new strategies to protect patients with glioma from the detrimental effects of stress on tumor progression.

## Supplementary Information

**Additional file 1.**

## Data Availability

All data generated or analysed during this study are included in this published article.

## Abbreviations

NE: Norepinephrine
EMT: Epithelial-to-mesenchymal transition
ADRB: β-adrenergic receptor
CNS: Central nervous system
GBM: Glioblastoma multiforme
SNS: Sympathetic Nervous System
CDS: Coding sequence
GFP: Green fluorescent protein
oligos: Oligonucleotides
DMSO: Dimethyl Sulfoxide
DAPI: 4′,6-diamidino-2-phenylindole
RIPA: Radio Immunoprecipitation Assay
PVDF: Poly vinylidene fluoride
TCGA: The cancer genome atlas
CGGA: Chinese Glioma Genome Atlas
WHO: World Health Organization
OS: Overall survival
