# Supplementary material for: Norepinephrine promotes glioma cell migration through up-regulating the expression of Twist1
Source: BMC Cancer. 2022 Feb 26;22:213. doi: 10.1186/s12885-022-09330-9 (PMC8882280; doi:10.1186/s12885-022-09330-9)
Supplement: Supplementary file 1 — Additional file 1. [file 12885_2022_9330_MOESM1_ESM.docx]

**Supplementary to:**

**Norepinephrine Promotes Glioma Cell Migration through Up-regulating the Expression of Twist1**

Xue Wang, Ying Wang, Fang Xie, Zi-Tian Song, Zi-Qian Zhang, Yun Zhao, Shi-Da Wang, Hui Hu, Yan-Shu Zhang, Ling-Jia Qian

**Supplementary Figures and Figure Legends**

**
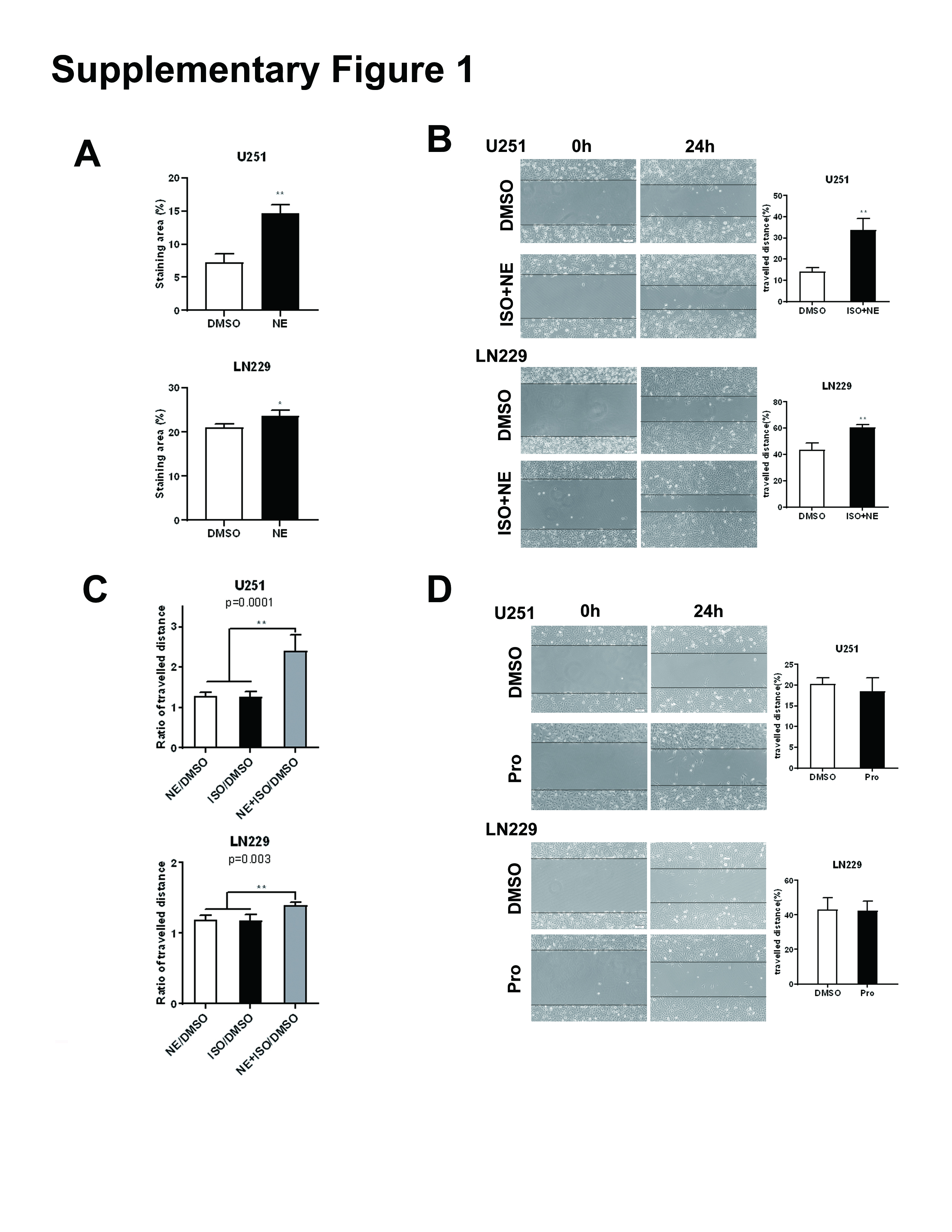
Supplementary Figure 1, related to Figure 1. NE promoted glioma cells migration via ADRB.** (A) The increased staining area percentage of transwell assays in NE-treated U251 (n=3, p=0.002) and LN229 (n=3, p=0.033) cells. Student’s t-test was used for statistical analysis. (B) Representative images and the higher travelled distances of wound healing in the NE+ISO groups of U251 (n=3, p=0.004) and LN229 (n=3, p=0.007). Scale bar, 50 μm. Student’s t-test was used for statistical analysis. (C) The ratio of NE+isoprenaline/DMSO was increased compared with that of NE/DMSO or isoprenaline/DMSO in the travelled distance (U251, p=0.0001; LN229, p=0.003). One-way ANOVA analysis was used for statistical analysis. (D) Representative images and no obvious difference were shown upon propranolol-treated U251 (n=3, p=0.449) and LN229 (n=3, p=0.892) cells. Scale bar, 50 μm. Student’s t-test was used for statistical analysis. * p < 0.05, ** p < 0.01.


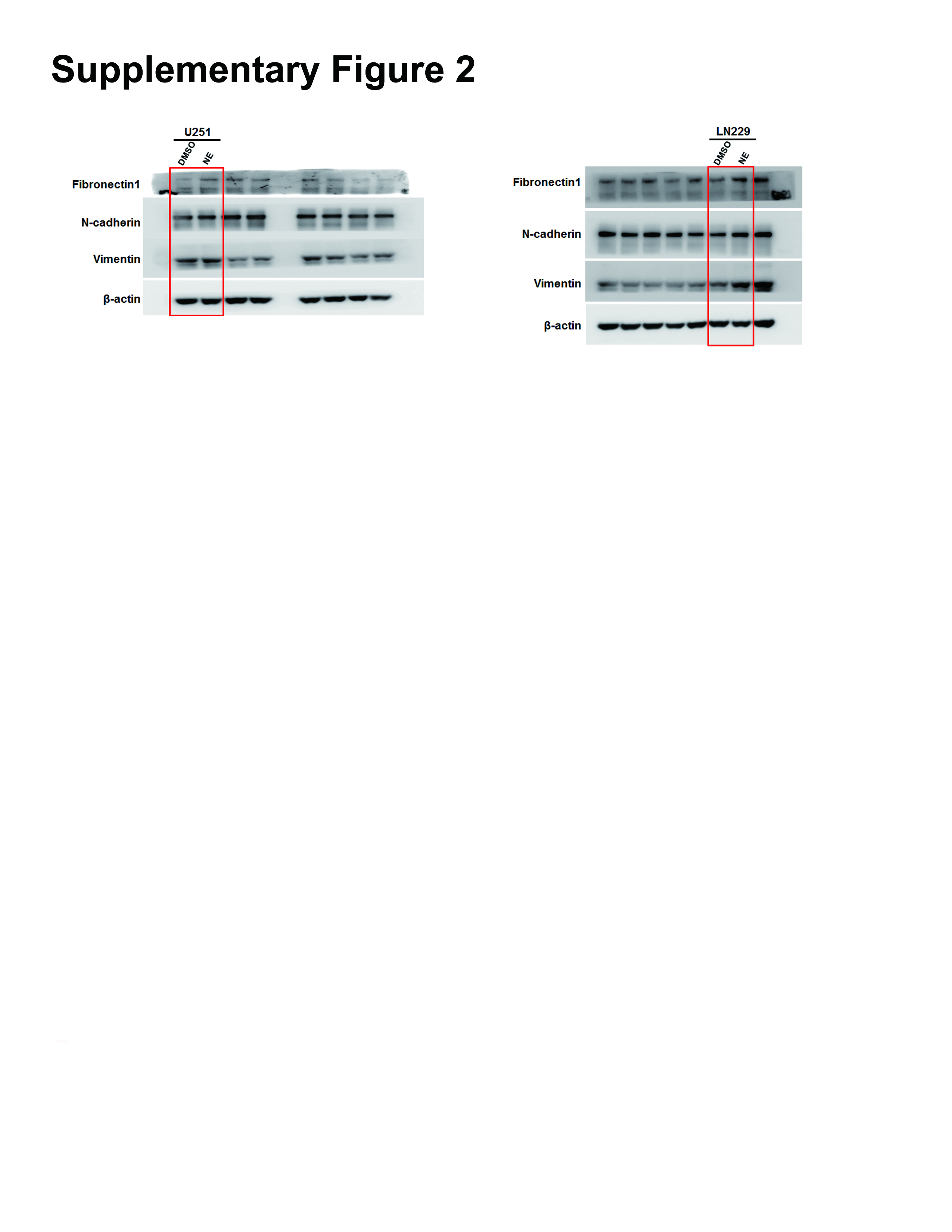


**Supplementary Figure 2, related to Figure 2. NE induced mesenchymal-like phenotype of glioma cells**. The original images of the blots in Figure 2D. The membrane was cut prior to hybridisation with different primary antibodies.


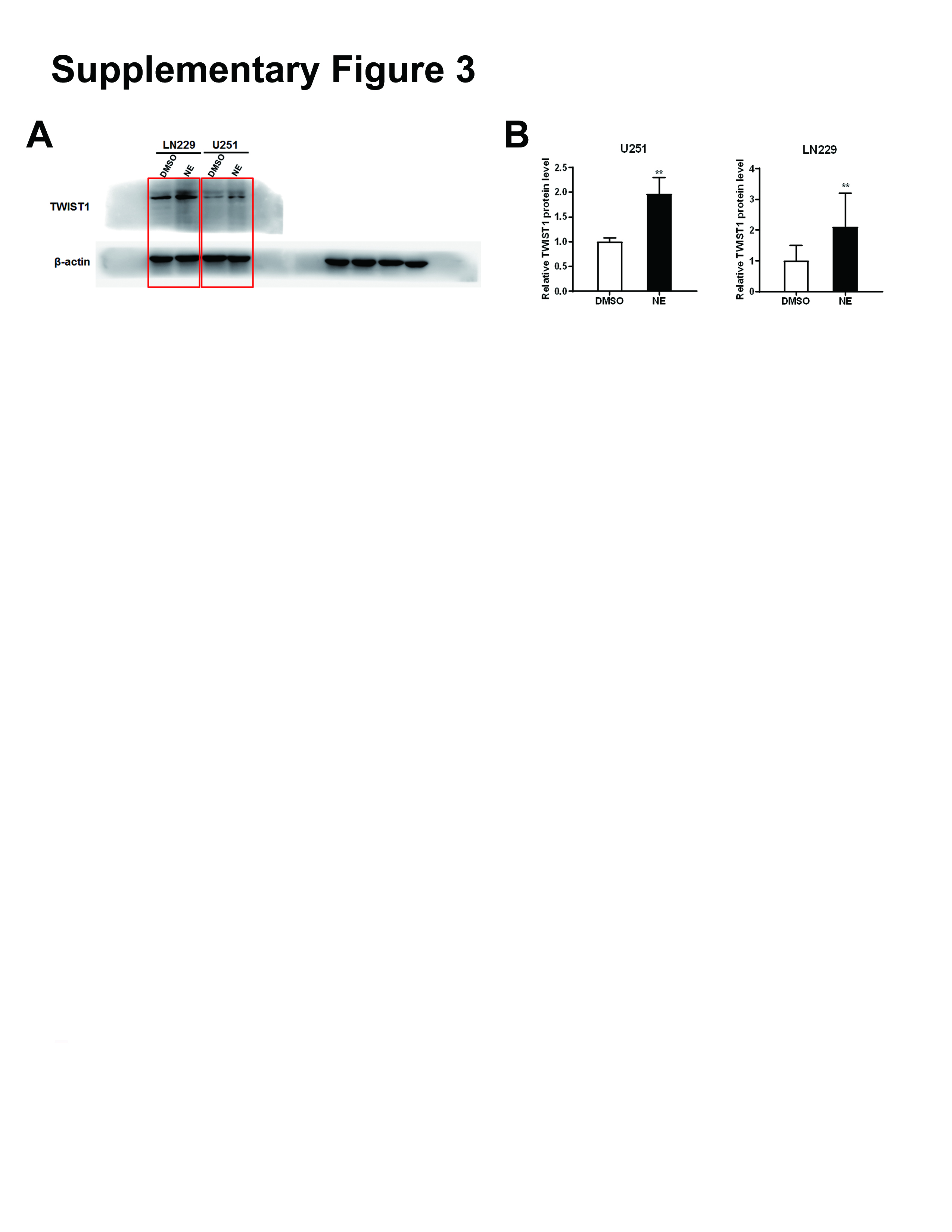


**Supplementary Figure 3, related to Figure 3. NE upregulated the expression of Twist1 in glioma cells.** (A) The original images of the blots in Figure 3B. The membrane was cut prior to hybridisation with different primary antibodies. (B) The level of Twist1 protein relative to β-actin was increased upon NE administration in U251 (n=3, p=0.007) and LN229 (n=3, p=0.005). Student’s t-test was used for statistical analysis.


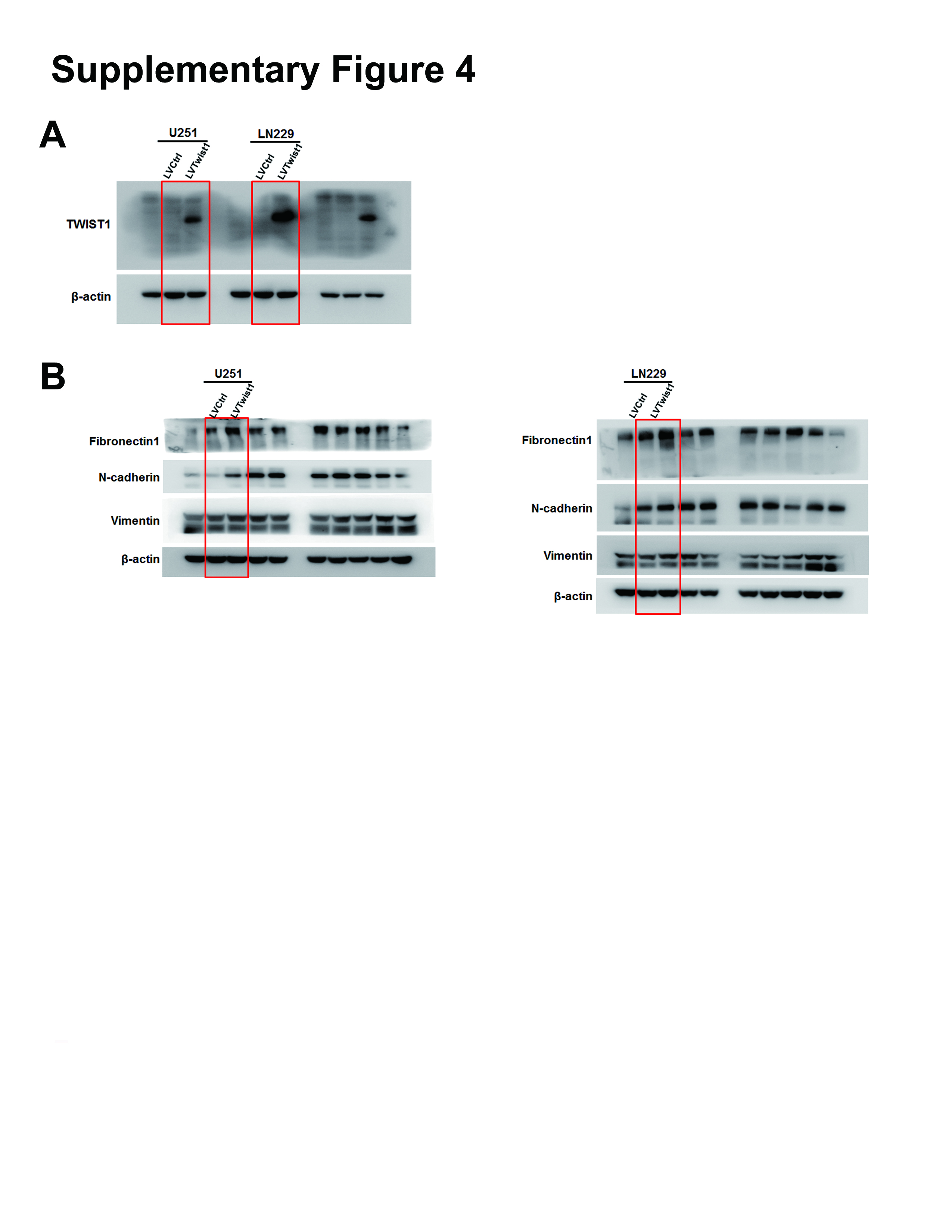


**Supplementary Figure 4, related to Figure 4. Twist1 overexpression facilitated mesenchymal-like phenotype and migration of glioma cells.** (A) The original images of the blots in Figure 4A&B. (B) The original images of the blots in Figure 4D. The membrane was cut prior to hybridisation with different primary antibodies.


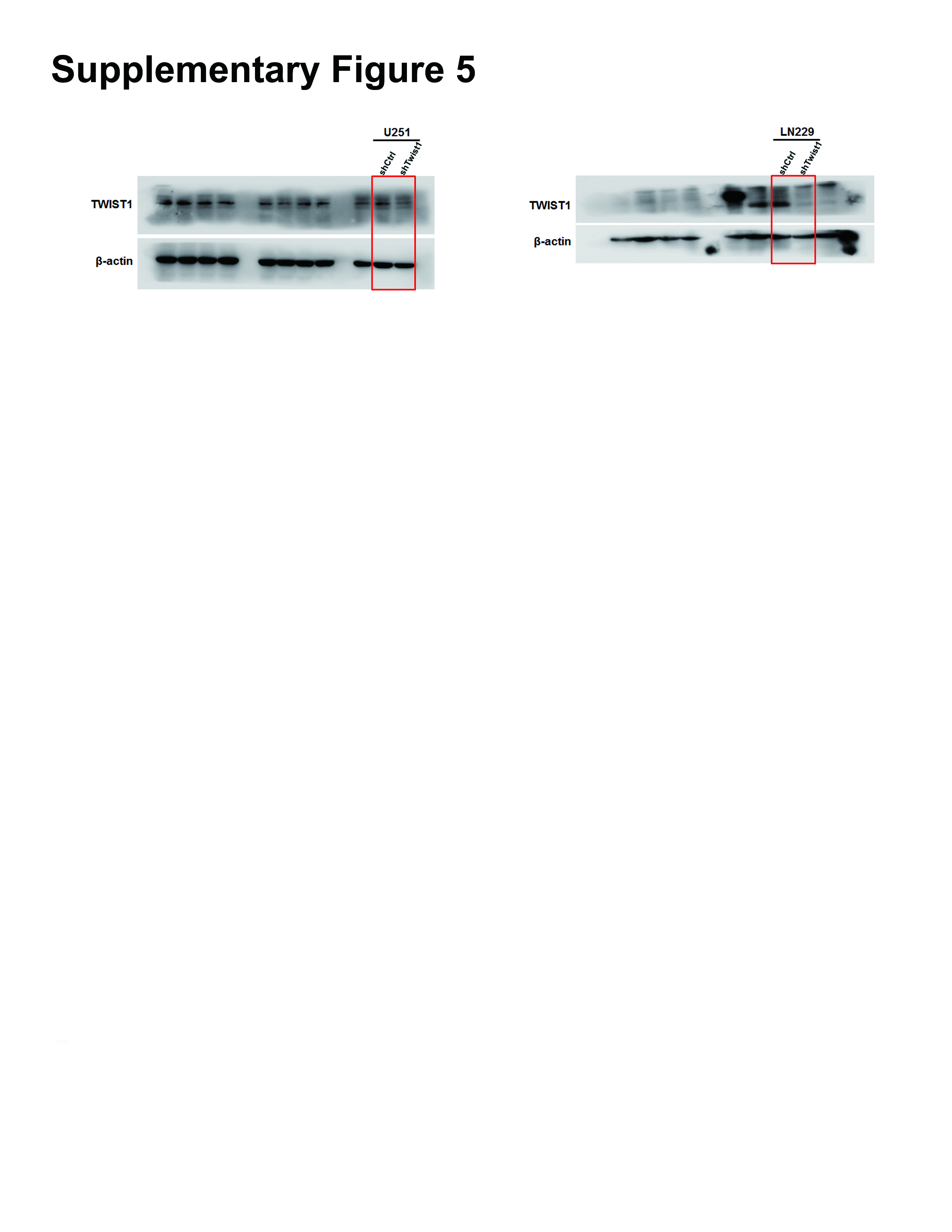


**Supplementary Figure 5, related to Figure 5. Twist1 knockdown suppressed NE-induced mesenchymal-like phenotype and migration of glioma cells.** The original images of the blots in Figure 5A&B. The membrane was cut prior to hybridisation with different primary antibodies.


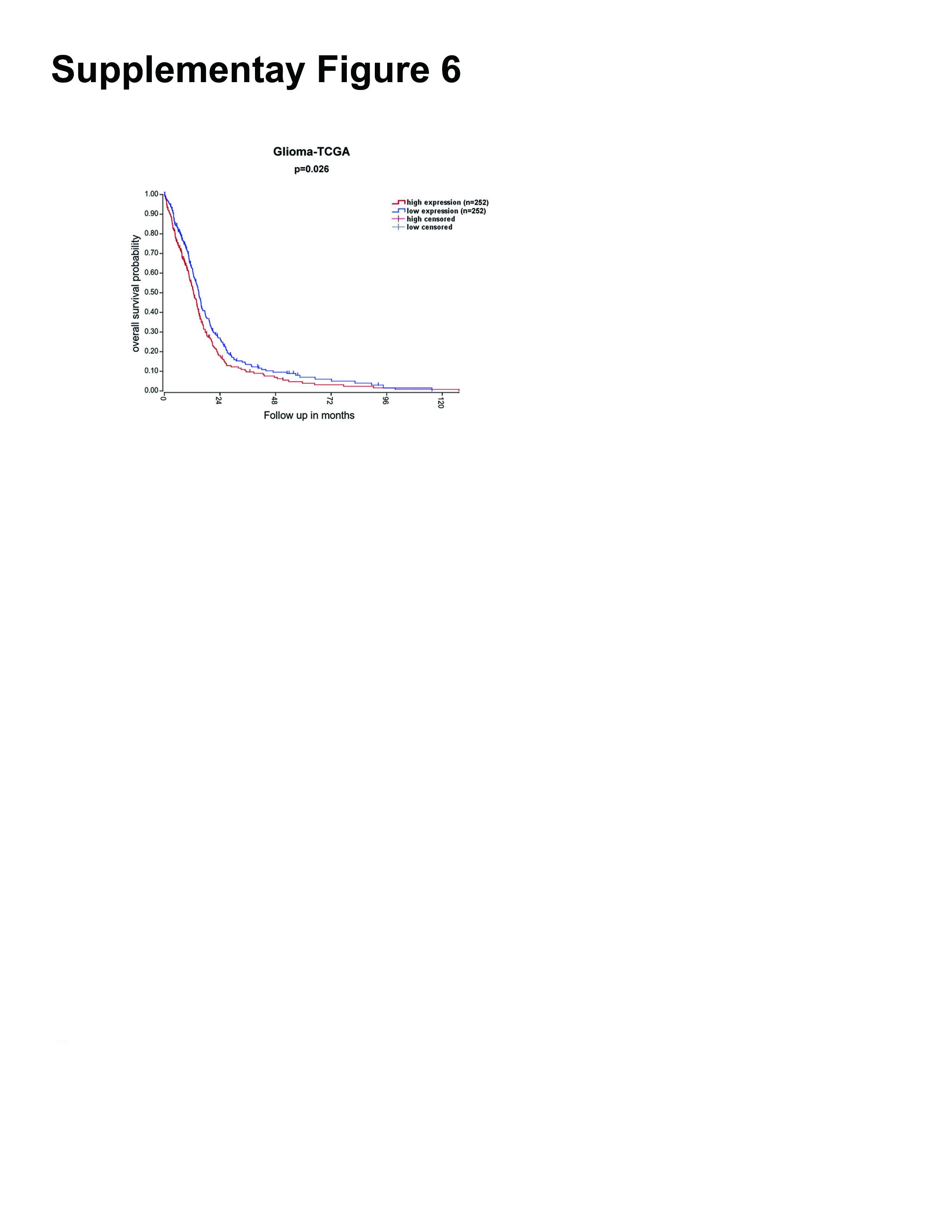


**Supplementary Figure 6, related to Figure 6. Expression and clinical correlation of Twist1 in glioma patients.** Kaplan-Meier survival analysis of patients from TCGA glioma database followed up with 120 months (n=504, p=0.026) and stratified by Twist1 expression. A lower survival probability in high Twist1 expressed patients compared with those patients with low Twist1 expression was revealed. Median Twist1 expression was used for stratification into Twist1 high expression and Twist1 low expression tumors.
